# From Intelligent Energy Management to Value Economy through a Digital Energy Currency: Bahrain City Case Study

**DOI:** 10.3390/s20051456

**Published:** 2020-03-06

**Authors:** Vangelis Marinakis, Haris Doukas, Konstantinos Koasidis, Hanan Albuflasa

**Affiliations:** 1 Decision Support Systems Laboratory, School of Electrical and Computer Engineering, National Technical University of Athens, 15780 Athens, Greece; h_doukas@epu.ntua.gr (H.D.); kkoasidis@epu.ntua.gr (K.K.); 2 Department of Physics, College of Science, University of Bahrain, Zallaq 32038, Bahrain; halbuflasa@uob.edu.bh

**Keywords:** digital energy currency, intelligent energy management, behavioural change, artificial intelligence, smart energy cities, AI-driven business model

## Abstract

The transition of the energy system into a more efficient state requires innovative ideas to finance new schemes and engage people into adjusting their behavioural patterns concerning consumption. Effective energy management combined with Information and Communication Technologies (ICTs) open new opportunities for local and regional authorities, but also for energy suppliers, utilities and other obligated parties, or even energy cooperatives, to implement mechanisms that allow people to become more efficient either by producing and trading energy or by reducing their energy consumption. In this paper, a novel framework is proposed connecting energy savings with a digital energy currency. This framework builds reward schemes where the energy end-users could benefit financially from saving energy, by receiving coins according to their real consumption compared to the predicted consumption if no actions were to take place. A pilot appraisal of such a scheme is presented for the case of Bahrain, so as to simulate the behaviour of the proposed framework in order for it to become a viable choice for intelligent energy management in future action plans.

## 1. Introduction

The worldwide demand for energy has raised concerns regarding depletion of resources, environmental and social impacts as well as difficulties in supply [1]. In recent years, the weight of taking action against climate change and implementing policies for effective energy management has started shifting towards communities of citizens. A step towards this direction is the Covenant of Mayors for Climate and Energy that tries to motivate the local authorities to adopt Sustainable Energy and Climate Action Plans (SECAPs) for the community they represent, committing 40% CO_2_ reduction target by 2030 [2]. Similarly, the Revised Energy Efficiency Directive requires utility companies to help their consumers use 1.5% less energy each year, especially within the framework of energy efficiency obligation schemes [3]. A more direct approach on engaging the energy consumers is the establishment of the energy cooperatives, which manage to make people jointly responsible for the energy system.

The above targets can be mainly achieved through appropriate energy efficiency measures that will be adopted by the big majority of the final energy end-users [4]. The issue of addressing the high demand for energy concerns scientists [5], and more research is focused on proposing actions for energy saving and intelligent energy management. One of the main drivers of energy consumption is the residential sector [6], which in Europe represents about 26% of the total energy consumed [7]. Therefore, one of the research topics that has been looked into is how to reduce energy consumption without however downgrading living standards [8,9] or user satisfaction [10]. Initially, researchers were focused mainly on increasing the energy efficiency in the building itself, by estimating the impact that alterations, such as improved insulation, could have on the total energy consumption [11], and the data-driven approach to optimal control of buildings cooling/heating system [12] and more.

Results indicated that the achieved saved energy could be enhanced if coupled with behavioural changes of the residents [13], with the potential savings of behavioural energy efficiency reaching up to 20% [14,15]. Although buildings’ occupants (energy end-users) seem to be gaining greater awareness of the value and need for sustainable energy practices, they do not behave in a more energy-conscious way. Behavioural change, in order to be triggered, should be accompanied by proper engagement, dissemination and maybe in some cases also by some kind of motivation to trigger efficient individual choices [16]. Proper incentives should be given to residents in order to foster that behavioural change. In the case of the residential building sector, residents need to be properly informed about the impact that behavioural changes can have on the environment. ICT-based solutions that exploit Artificial Intelligence (AI) algorithms and Internet of Things (IoT) technologies can contribute significantly to energy saving [17,18], by motivating and supporting behavioural change of the buildings’ occupants [19]. In recent years, AI-based physical and virtual platforms have been presented to develop sustainable smart energy cities [20].

The notion of energy conservation by motivating energy end-users to change their behaviour patterns is not new. Already from 1998 in ETH Zurich, the notion was introduced with the 2000-watt society that aimed to reduce overall average primary energy usage to no more than 2000 watts by the year 2050, without lowering standards of living [21]. The notion of energy efficiency per capita is already in place [22], where all the developed energy-related monetary instruments that are available are presented.

What is new is a feedback loop between nature and economy, in terms of developing a more stable unit of value, attached to the planet’s natural sustainable resources, namely the kWhs saved.

Recently, various alternative trading schemes have been implemented in local ecosystems and smart energy cities, which endeavour to create sustainable economic and social improvement. In this context, the concept of energy-currency has been created, which suggests that energy can be used as an alternative and/or complementary currency in urban settings while acting as a compelling incentive for meeting energy targets [23]. Although most of the recent efforts address environmental issues, they overlook the energy efficiency sector. The connection between energy-saving behaviour and coins earning has to be studied and formalised [24].

The purpose of this paper is to present the ATOMcoin framework, introducing a formula for the calculation of the daily rate of the digital energy currency. Moreover, the pilot appraisal of the proposed framework in the case of Bahrain is presented and discussed. The proposed currency is founded on the basis of approaching energy directly as a monetary entity and using it in the process of providing occupants with the financial incentives to trigger energy-saving actions.

The current study builds upon the findings of this previous study [19] and addresses its limitations. This study looks into the case of residents changing their behaviour patterns regarding energy consumption if further motivated by a reward scheme that will provide real-time feedback on energy-saving actions. Human behaviour is one of the most important factors regarding energy saving; hence introducing a framework that involves this element can result in a substantial reduction of the energy consumed in the residential sector. The exact calculation of the amount of energy saved can be achieved with the aid of advanced information technology.

In the sections below, the main concepts, the methodology, a numerical example and this study’s contribution are presented. The rest of the paper is organised as follows. In Section 2, a theoretical approach is presented concerning the entities that could benefit from such a scheme as long as the technologies required. Specific examples are referenced about similar approaches. In Section 3, the innovative scheme of the proposed currency is thoroughly presented, while in Section 4 it is used in a case study from Bahrain simulating a realistic implementation. In Section 5, the contributions and conclusions of the current study are discussed respectively.

## 2. Structure of the Framework

### 2.1. Related Works

A number of tools have already been developed, aiming to provide solutions to increase energy efficiency, focusing on more traditional approaches on energy conservation. Origami Energy (London, UK) [25], SMARKIA (León, Spain) [26] and PlotWatt (Durham, NC, USA) [27] aim to assist companies by providing platforms that focus on efficient usage of energy resources through monitoring. A similar approach is used by NUUKA (Helsinki, Finland) at the building level [28]. Plugwise (Sassenheim, The Netherlands) [29] and OPTIWATTI (Espoo, Finland) [30] mainly focus on temperature control to decrease consumption regarding heating and cooling. Loop Energy Saver (Woodbridge, UK) [31] constitutes an energy-saving assistant at the household level, that provides advices related to energy consumption.

These tools provide an important step towards energy efficient behaviours through monitoring. Some of them focus on data entry about energy consumption or home appliances, and allows the users to calculate the energy costs associated with their everyday activities to see where they are spending the most on electricity. Other applications, using smart meters, allow the users to see how much electricity they have been using throughout the year, month or day and gain insights into when they use electricity and how much it costs. In most of these cases, the use of these applications requires product installation.

As a result, the aim of these applications is to capture the building occupants’ attention and provide them with the information they need to manage their energy use properly. However, this process requires a continuous and non-stop engagement from the energy end-users, who usually have the tendency to lose interest on an app after a while, if not adequately motivated [32]. The proposed framework attempts to address this issue by incentivising the energy efficiency process through a digital energy currency, thus providing an additional motive for the end-users to save energy. A detailed analysis of the structural blocks of the proposed framework is presented in the following paragraphs.

### 2.2. Components of the Proposed Framework

**Figure 1:**
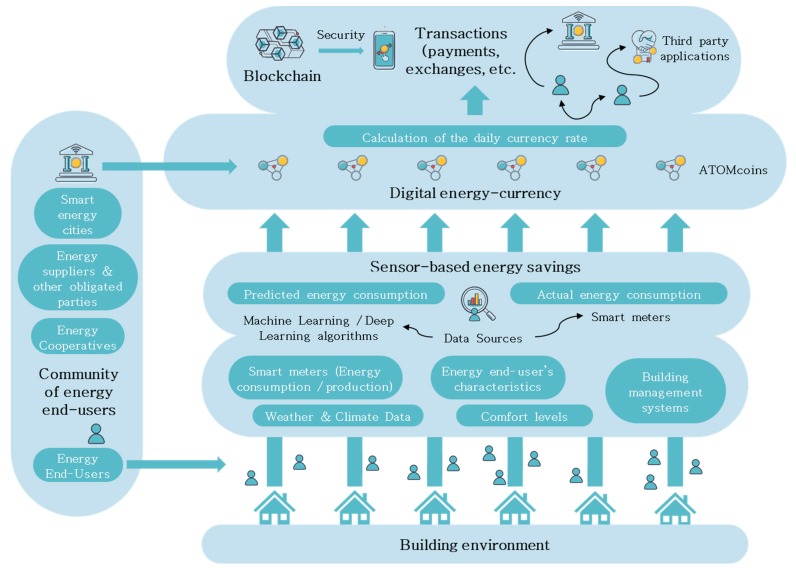
Components of the proposed framework.

The proposed framework consists of several components which are essential for its implementation (Figure 1), namely: (1) Building environment; (2) community of energy end-users which is managed by an authority (smart-energy city); (3) use of sensors, such as smart metering devices and other data sources to calculate the actual energy savings; (4) digital energy currency.

The proposed reward scheme could be implemented by the local and regional authorities, as well as energy suppliers, utilities and other obligated parties, or even energy cooperatives. AI algorithms, like neural networks, machine learning and deep learning can be combined with sensors to identify energy consumption patterns at the household level, thus leading to the calculation of the actual energy savings. These savings are then converted to ATOMcoins and distributed to the end-users according to their energy efficiency performance. Transactions, like payments and exchanges, based on the digital energy currency can be monitored and registered on secure databases, exploiting the opportunities provided by blockchain technology.

### 2.3. Building Environment

These consist of building occupants and the integration of the buildings to the community. In our approach, the buildings examined are solely residences. However, in a similar scheme, any type of establishment would possibly be included. Specifically, buildings of the public sector often constitute a substantial share of a country’s whole building use [33]. The accomplishment of the city’s energy-saving goal is gravely dependent on the behaviour of the residents.

Energy savings are gravely dependent on human behaviour, since it has been shown that a big portion of electrical loads in residential areas are very sensitive to people’s actions [34]. Nevertheless, it has been observed that human behaviour is related to the internal and external conditions as well as the physical features of the buildings. For example, some of these external factors that affect a household’s energy consumption are the outside temperature, the possible absence of sunshine as well as the cost of energy. Consequently, affecting the human aspect, in order to achieve energy saving, can become challenging.

One of the most dominant ways of affecting the aspect above is the development of Demand-Response Mechanisms (DSMs). For example, Li et al. (2014) presented an occupant- engaged demand response strategy for building automation, in which residents are encouraged to adapt their energy consumption in response to incentive opportunities designed by faculty managers [35]. This study, among many others, concludes that human behaviour needs to be taken into consideration when designing any energy-saving framework.

### 2.4. Community of Energy End-Users

#### 2.4.1. Smart Energy Cities

This feature characterises the urban setting where the scheme may be implemented in. For example, it can be a smart-energy city which endeavours to reduce its energy consumption by setting an annual goal. Consequently, the level of energy use for that particular year without the participation in the said scheme must be calculated. This value sets the Business-As-Usual (BAU) scenario, which will be compared with the actual amount of energy consumed that specific year in order to evaluate the efficacy of the program. This solution comes in accordance with the Covenant of Mayors, where each local or regional authority is required to develop a Sustainable Energy (and Climate) Action Plan. By using a small budget for this scheme instead of, or in parallel with, other actions, a city could achieve the targets of the domestic sector which plays a significant role on the total consumption [36].

#### 2.4.2. Energy Suppliers and Other Obligated Parties

The proposed framework can also be used as a reward program by an energy supplier utility for its customers. This can be beneficial for both the end-user, who receives the reward in the form of the digital currency, but also for the utility within the framework of the Energy Efficiency Obligation Schemes (EEOSs). Under the Energy Efficiency Directive (2012/27/EU and its update on 30 November 2016), energy distributors or retail energy sales companies have to achieve 1.5% energy savings per year to final consumers. This can be mainly achieved through appropriate energy efficiency measures that will be adopted by the large majority of the final energy end-users. However, since traditional methods such as flyers have a great cost without sensitising a large portion of the receivers, it might be efficient to raise the awareness by using reward programs such as the currency presented. Therefore, goals set by commitments regarding energy efficiency could be fulfilled avoiding penalties that might take place.

On the other hand, as it is shown in Hassan (2017), peak demand can be very costly for energy suppliers-utilities since it requires more capacity [37]. By rewarding the customers who save energy, the peak downshifts to result in a positive financial effect on the utilities, despite the fact that the utilities’ main objective is to maximise profit from energy sold [38]. This positive effect regardless coupled with of the obligations mentioned above could justify the necessary effort required by a utility to trigger energy efficiency [39].

#### 2.4.3. Energy Cooperatives

An energy cooperative may choose to produce energy which offers a simple business plan for the cooperative to be both financially successful and beneficial for its participants, but it can also play an important role on the transmission, distribution of energy and other forms of activities related to the energy sector. Although energy efficiency has a great potential for energy savings, different types of business plans need to be created [40], making it, accordingly, more difficult for cooperatives to engage primarily on such activities. In fact, as shown by Debor (2014) [41] cooperatives focusing on the production of energy in Germany constituted more than 60% of the total cooperatives. In Europe, there has been an increasing interest on energy cooperatives expressed by the initiative of REScoop.eu which is a network uniting projects into a federation that currently include around 1500 cooperatives, representing an estimation of 1,000,000 European citizens. The purpose of REScoop is to promote the idea of cooperatives in Europe by supporting energy democracy and a bottom-up approach on the production of decentralised energy where anyone can participate in the governance of the energy system.

A digital energy currency based on energy savings can be used inside an energy cooperative to motivate the members into being more efficient in the use of energy, thus creating the opportunity for establishing business plans for energy cooperatives with the focusing mainly on energy saving. Energy cooperatives, as a mechanism, have the ability to provide opportunities to promote and expand existing ideas such as “prosumers” and net-metering by organising atoms into teams that could even create micro-grids in order to take active part on the green transition. An energy currency could unite all these ideas with ICT and blockchain into building a new model for the energy system.

### 2.5. Sensor-Based Energy Savings

The third component is the use of ICT equipment, which can be any type of smart metering devices, such as real-time energy monitoring, reporting and automation devices [42,43], as well as building management systems. In order to generate the daily BAU scenario for each household, the forecasted values of energy consumption are required. As a result, these sensor-based data combined with other data sources (weather and climate data, comfort levels and energy end-user’s characteristics) are necessary for the forecasting of energy consumption and calculation of the actual energy savings.

In Marinakis and Doukas (2018) [44], an advanced IoT-based system for intelligent energy management in buildings introduced and could be utilised in this case, where the residents are made aware of their exact energy consumption, their goals as well as whether and how their energy saving is translated to digital coins. An application is proposed for intelligent energy management, linking big data to intelligent energy management [45].

More specifically, in calculating BAU scenarios, a lot of factors are taken into consideration, such as the weather, the indoor temperature, etc. These data are inserted into machine-learning algorithms to create more robust scenarios involving multiple variables. Artificial neural networks play a significant role in energy demand forecasting, employed in 40% of planning models [46] for a variety of sectors, like transport energy demand [47], cooling energy load [48] and energy forecasting for short term periods [49,50] and midterm periods [51]. Different machine-learning approaches like Support Vector Machines (SVMs) were compared showing promising results [52]. Jain et al. (2014) used the SVM method to forecast the energy consumption of buildings showing that sensor-based forecasting methods could lead to effective models [53], especially coupled with wireless sensor networks [54] that could provide the necessary data [55]. Recently, deep reinforcement learning was introduced for the optimal energy management [56,57] of microgrids [58] and buildings [59], with emphasis on predicting models [60,61]. Lee et al. (2019) proposed an energy consumption prediction system based on deep learning with edge computing [62]. Lu and Hong (2019) proposed a demand response framework using reinforcement learning to calculate the rate of an incentive to the customers for balancing energy fluctuations [63]. All the above contribute to the prediction of the daily energy consumption of each household, which plays a crucial role in the awareness of the citizens about the achieved energy savings keeping in consideration that if the feedback is to be provided to the citizens, the above process must happen in real-time. In this way, residents will be able to adjust their behaviour to achieve the desired goal.

### 2.6. Digital Energy-Currency

The available energy-currency schemes vary on the technology used, but mostly on the necessity they try to address. There are several initiatives that use the blockchain technology to establish a platform for energy trading and smart contracts, such as Power Ledger [64] with a market cap of more than 40 million dollars, Electrify.Asia [65], and Suncontract [66]. Robotina [67] is a platform which combines blockchain with IoT in order to help its users to save energy. In order to establish the BAU scenario, it uses AI with machine learning to forecast the expected consumption based on historic data and weather reports. These platforms offer a token that can be rewarded to the users and can be monetarized inside the platform (e.g., software, hardware and other services on the platform). WePower [68] and Bittwatt [69] are also platforms for energy trading and smart contracts which rewards the producers of green energy on the concept of 1 token = 1 kWh. The same concept is also used by SolarCoin [70], which enables the producers of solar energy to register their production on a platform and be rewarded with 1 coin per 1 MWh. The field of energy currencies expands as there are more initiatives creating forms of cryptocurrencies linked with energy currently with a market cap lower than 1 million dollars. Nevertheless, most of the coins presented are strongly dependent on the platform in which they are implemented, causing limitations in the extend in their use.

The concept of energy currencies also received attention from the academic field to solve the problems addressed above. In 2010, the idea of Ergo was introduced by Sgouridis and Kennedy (2010) [23] as a progression of the Trading Energy Quotas [71], which was a credit system representing an energy unit for all the activities on the energy sector. On a different axis, NRGCoin presents a virtual currency which rewards producers of energy from renewable sources with the cooperation of a DSO [72]. The main concept examined is that it is possible for both the producer and the consumer to have financial gain by trying to level the amount of energy produced and consumed. However, the value of such a currency in the market is left uncovered since the calculations only involve the internal phenomena of the system.

In this context, a digital energy-currency is introduced to incentivise behavioural energy efficiency. Energy end-users are able to earn ATOMcoins by reducing or shifting their energy consumption. For every 1 kWh reduced, the end-user earns the respective ATOMcoins, which can be used to decrease final energy cost, direct exchanges etc. In Section 3, the calculation of the daily currency rate of ATOMcoin is presented step-by-step.

It is noted that Distributed Ledger Technology could be exploited to guarantee and track the use of ATOMcoins and how the owners are sharing them. The automation of transaction records and the authorisation based on the agreements can be done using smart contracts to effectively manage the needs and to simplify interactions. Indeed, ICT paved the way for implementing technologies such as blockchain that might have a different primary use [73], but that could effectively create solutions in different areas [74,75]. One of these is the energy industry where blockchain projects and start-ups start to emerge [76,77]. An important example of such a case is the Brooklyn Microgrid [78,79] which uses a blockchain platform to support energy transactions. However, the usage of blockchain comes at a cost regarding energy consumption. The mining of a high number of existing cryptocurrencies is based on the proof-of-work algorithm, and as the mathematical problems required by the protocol for hashing become more complex, so does the energy required to solve them [80]. In order to deal with this problem, non-competitive algorithms can be used, such as, proof-of-stake [81]. Since most of the projects in the energy industry are about energy efficiency, it is important to take into account the energy consumption when implementing blockchain technology.

## 3. Calculation of the Daily Currency Rate

### 3.1. Definitions

Primarily this scheme is designed for an urban environment in the form of a community. Specifically, it could be a smart-energy city, but it could also be a utility or any other entity that aims to create an energy-saving scheme. Towards this direction, external funding needs to be granted by the community in order to achieve its goal. This capital can be invested in various ways. For example, it can be invested to enhance the building’s insulation, leading to the reduction of the total energy consumption. Instead, in the said scheme, the capital is invested in supporting the implementation of the proposed framework and dispersing value in the digital coins considered in this project. Particularly, the framework will be implemented for a specific number of days (i.e., k number of days) and a specific number of households (for instance, N residences). Furthermore, every household has available, real-time information on its actual energy consumption, its daily energy goals as well as the way in which their actions are translated to energy savings, with the aid of ICT equipment. Hence, each household is able to calculate the amount of ATOMcoins claimed. In this scheme, every day i, each household j obtains ESij digital coins which are equal to the amount of kWh saved.

### 3.2. Limitations

The proposed framework in this paper addresses some of the limitations presented by Marinakis et al. (2018) [19].

- The budget, on the case it remains fixed, is not dependent on the achieved savings. For instance, if there are few savings (${\mathrm{ES}}_{\mathrm{ij}})$, the same capital will be distributed ($B_{i}$), but it will result in a small amount of coins, which will have a higher currency rate, as the followings calculation illustrates:
  (1) $$\underset{{\mathrm{ES}}_{\mathrm{ij}}\to 0}{\lim}\left(\frac{B_{i}}{{\mathrm{ES}}_{\mathrm{ij}}}\right)\;=\;\infty$$
  Therefore, a high amount of money would have been spent without accomplishing the desirable outcome, even causing an undefined ratio on the case that there are no savings on a specific date.
- On the contrary, if there is a high amount of energy saved, the currency rate will be extremely low, since a lot of coins will be generated:
  (2) $$\underset{{\mathrm{ES}}_{\mathrm{ij}}\to \infty }{\lim}\left(\frac{B_{i}}{{\mathrm{ES}}_{\mathrm{ij}}}\right)\;=\;0$$

In fact, as shown in Calculation (2), an increase in the energy saved in high amounts might cause the ratio to tend to zero and lose all its value.

Therefore, due to these limitations, a lot of residents may get discouraged and stop saving energy since they will not be rewarded with a more valuable and socially-just digital currency. For example, when the participants invest a lot of effort in saving energy but at the same time the rate is low the reward program fails its purpose. Finally, the methodology used to calculate the ratio caused each coin to have a specific value when distributed at first and keeping that value for as long as it was in the market, which caused the coin to lose trading value.

### 3.3. Adopted Approach

In this approach, the value of each ATOMcoin may fluctuate daily, since it is defined by a variety of parameters. The daily currency rate C_i_ of the generated coins at day *k* is expressed in €/coin and calculated as shown below:

(3) $$C_{k}\;={\;p}_{\mathrm{kWh}}*\mathrm{reg}*\left(0.5*\frac{\sum_{i=1}^{k}\left\{\sum_{j=1}^{N}\left[{\mathrm{ES}}_{\mathrm{ij}}-{\left(\mathrm{cc}-\mathrm{cb}\right)}_{i-1}\right]\right\}}{\sum_{i=1}^{k}\left[B_{i}+\mathrm{reg}*e-{\mathrm{ratio}}_{i-1}*{\left(\mathrm{cc}-\mathrm{cb}\right)}_{i-1}\right]}\;+\;0.5*\frac{\sum_{i=\;k-5\geq 0}^{k}{\mathrm{ES}}_{\mathrm{ij}}}{\sum_{i=\;k-5\geq 0}^{k}B_{i}}\right)$$

In the above formula, $p_{\mathrm{kWh}}$ denotes the current cost of kWh expressed in €. Moreover, e, cc and cb stand for the excess from predicted of kWh consumed which will be penalised, coins cashed out and coins brought. respectively. The last two variables give the participants the chance to sell the earned coins back to the central authority before the completion of the scheme, and other users to buy these coins. Direct exchanges between participants do not affect the calculations.

In the parenthesis, the first fraction represents the long-term changes in the scheme since it takes into account all the past data, the penalties and the coins cashed out or brought again. The second fraction represents the short-term changes, calculating only a specific number of past days, in our case the data of the previous five days. The two fractions are weighted equally in order for the last five days to be fully calculated, while the other previous days receive lower weights. This is to ensure that the ratio has some stability provided by the long-term factor, as well as the necessary volatility from the short-term factor to create an opportunity for profit. The main reason we prefer an additive model is because the short-term factor might occasionally receive very small values. For example, if there is a very cold weather for five consecutive days energy consumption will rise due to heating so the energy saved will be minimised, causing the ratio to tend to zero in a multiplicative model. In addition, reg is calculated using the following formula:

(4) $$\mathrm{reg}\;=\;\frac{\mathrm{Total}\;\mathrm{Budget}}{\mathrm{Energy}\;\mathrm{Saving}\;\mathrm{Goal}}\;\mathrm{in}\;(€/\mathrm{kWh})$$

The regulator (reg) provides the expected number of euros spend to save 1 kWh. Therefore, if the amount of kWh saved and the euros are balanced, as expected, the product of reg with the amount in the parenthesis will tend to 1. Consequently, in the typical case scenario, where the budget is used to save the necessary amount of energy, the currency rate will balance in the current cost of kWh ($p_{\mathrm{kWh}}$), which means that for every kWh saved, the household will claim an extra kWh in digital coins. However, the authority running the scheme might choose a different reward for every kWh saved by replacing $p_{\mathrm{kWh}}$ with another value. Moreover, in Formula (3), $B_{i}$ symbolizes the budget distributed at day i which is the share of the available capital allocated on that day. $B_{i}$ can fluctuate daily to avoid seasonal effects added by consumption, but it can also remain steady. In the case that it remains fixed it can easily be calculated using the following formula:

(5) Βi = BTk in €

Furthermore, each household j has collected $m_{i}$ amount of ATOMcoins after participating in this framework for k days:

(6) $$m_{j}\;=\;\sum_{i=1}{\mathrm{ES}}_{\mathrm{ij}\;}\;\mathrm{in}\;\mathrm{ATOMcoins}$$

Respectively, the monetary gain of each household j on day k in this scheme can be determined as shown:

(7) $$g_{\mathrm{kj}}\;={\;C}_{k}\;*\;\sum_{i=1}^{k}\left({\mathrm{ES}}_{\mathrm{ij}}\right)\;\mathrm{in}\;€$$

This monetary gain expresses the amount of € household j will receive in case it cashed out its coins on day k.

In conclusion, after the completion of the framework, each household j has claimed $m_{i}$ ATOMcoins. This digital currency can be exchanged in every transaction which allows the use of ATOMcoins.

### 3.4. Contributions

The existing fraction of the initial equation [19] was inverted, placing the energy saved on the numerator:

(8) $$C_{k}=\frac{B_{k}}{\sum_{j=1}{\mathrm{ES}}_{\mathrm{kj}}}\to {\;C}_{k}=\frac{\sum_{j=1}{\mathrm{ES}}_{\mathrm{kj}}}{B_{k}}$$

This causes the ratio to follow the trend of the energy saved by the participants, so when the currency is popular, and the users want to earn it by saving more energy, the ratio increases, thus increasing its value. This is in accordance with other digital currencies, such as Bitcoin [82], where an increase in public interest came along with their value growing rapidly [83].

As it is already mentioned, the existence of both a long-term and a short-term factor make the coin more reliable by combining steadiness with volatility, since increased volatility creates instability [84], thus not been in accordance with efficient markets [85]. To deal with this issue, past data have been introduced in Equation (10), with the two described factors equally weighted:

(9) $$C_{k}\;=\;0.5*\frac{\sum_{i=1}^{k}\left\{\sum_{j=1}^{N}\left[{\mathrm{ES}}_{\mathrm{ij}}\right]\right\}}{\sum_{i=1}^{k}\left[B_{i}\right]}\;+\;0.5*\frac{\sum_{i=\;k-5\geq 0}^{k}{\mathrm{ES}}_{\mathrm{ij}}}{\sum_{i=\;k-5\geq 0}^{k}B_{i}}$$

Two important changes also introduced were the penalty for excessing the target for consumption and the opportunity for trading the currencies. The idea behind this penalty is that by consuming more energy than anticipated the scheme is set back, since this excess should be balanced on later days. The value of reg is used because it gives us an estimation on how much money we need to pay to save 1 kWh generally and, in this case, the excess. It is important to choose a constant value in order to avoid penalties that might be financially unbearable. In Equation (10), variables for the penalty and trading have been introduced to include the three different categories of economic incentives as described by Sunstein (1999) [86]; subsidies for beneficial behaviour, penalties for harmful behaviour and trading of rights:

(10) $$C_{k}\;=\;0.5*\frac{\sum_{i=1}^{k}\left\{\sum_{j=1}^{N}\left[{\mathrm{ES}}_{\mathrm{ij}}-{\left(\mathrm{cc}-\mathrm{cb}\right)}_{i-1}\right]\right\}}{\sum_{i=1}^{k}\left[B_{i}+\mathrm{reg}*e-{\mathrm{ratio}}_{i-1}*{\left(\mathrm{cc}-\mathrm{cb}\right)}_{i-1}\right]}\;+\;0.5*\frac{\sum_{i=\;k-5\geq 0}^{k}{\mathrm{ES}}_{\mathrm{ij}}}{\sum_{i=\;k-5\geq 0}^{k}B_{i}}$$

As a final step towards the proposed Formula (3), the variables $p_{\mathrm{kWh}}$ and reg are inserted into Equation (10) to secure that the currency will fluctuate around the value of the commodity represented, in order to reduce the risk for cybercriminal activity that is associated with the rise of digital currencies [87]. The product of the variable insertion is Formula (3), concluding the derivation process that provides valuable insights on the new concepts that are considered from the suggested approach. However, the manner that the formula will be used also contributes to the initial approach.

More specifically, the daily budget is not fixed in the current study, so the budget destined for days with no savings is allocated to days in which great savings are occurring. To achieve that, the daily budget is distributed according to the projected daily-demand peak, as it is expected that in days with higher consumption levels, the potential for energy saving also increases.

Finally, different categories of end-users can be considered according to their energy-saving status and get more representative numbers. In addition, the use of ICT equipment combined with the installation of photovoltaic cells on residences’ rooftops can provide households the ability to sell energy to other households and obtain digital energy currency. This will commence an era of peer-to-peer (P2P) energy market, which will benefit the grid since energy will be distributed in smaller distances. Moreover, ICT can provide real-time feedback in the process and provide data on the way that residents benefit from their digital currency income and data for the currency’s circulation in the market. In this manner, the government will be able to enable the use of digital coins in various transactions.

## 4. Pilot Appraisal

### 4.1. Bahrain City Case Study

The proposed scheme was tested in the case of Bahrain, which is a small island in the Gulf Region across Saudi Arabia. The population is 1,494,090 people according to the 2017 census, and the total energy consumption is approximately 16,559 GWh. Fifty percent of this amount is consumed in the residential sector [88]. Furthermore, the energy mix of Bahrain is heavily dependent on fossil fuels thanks to the rich natural oil and gas deposits located in the region. Hence, the regulation of energy consumption results in the mitigation of climate change.

Specifically, the government of Bahrain intends to reduce the consumption of electricity by 6% until 2025. In order to accomplish the best possible result, it is assumed that every household in Bahrain participates in the proposed scheme. This means that the total number of households contributing to the energy-saving target is approximately 250,000 [89]. Consequently, the anticipated savings were calculated to be 539.8 GWh, which represents the 6% of the total consumption of 2018 for the domestic sector, which was estimated to be approximately around 8997 GWh. This consumption was distributed to the households according to the number of members they include [90] with the assessment that the per capita consumption of electricity is around 5608 kWh. This distribution creates the target for each household per day, where the daily allocation was calculated based on statistics from Bahrain Electricity and Water Authority.

### 4.2. Results

After the BAU scenario, in order to calculate the daily energy savings of the households, the real consumption should be simulated. For this test, we calculated a typical case scenario where the real consumption follows a normal distribution of around 94% of the expected consumption, with a standard deviation of 10%. In this case, a capital of $50,000,000 will be distributed in the form of ATOMcoins in order to encourage residents to reduce their energy consumption. According to the formulas above, the value of reg was equal to 0.093 $/kWh and the value of p_kwh_ to 0.0783 $/kWh, which in our case is the average price of electricity in Bahrain converted into dollars.

This implementation of the framework resulted in the mitigation of the energy consumed by 691.5 GWh. This amount was higher than the anticipated savings which lead to the assumption that the scenario of ~N (μ = 94%, sd = 10%) is slightly optimistic. However, the proposed scheme shows great potential since it is possible to surpass the target of the Bahraini government without causing financial problems.

**Figure 2:**
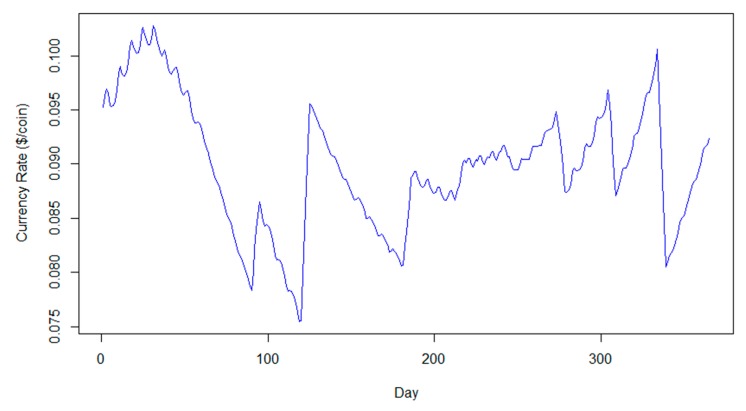
Daily currency rate for 365 days.

The average currency rate was calculated around c_average_ = 0.089 $/coin higher than the current cost of energy; hence, the regulation of energy consumption can become financially beneficial for the residents, since the ATOMcoin generated by saving 1 kWh of energy will have a higher value than the cost of 1 kWh of energy purchased (Figure 2).

### 4.3. Sensitivity Analysis

**Figure 3:**
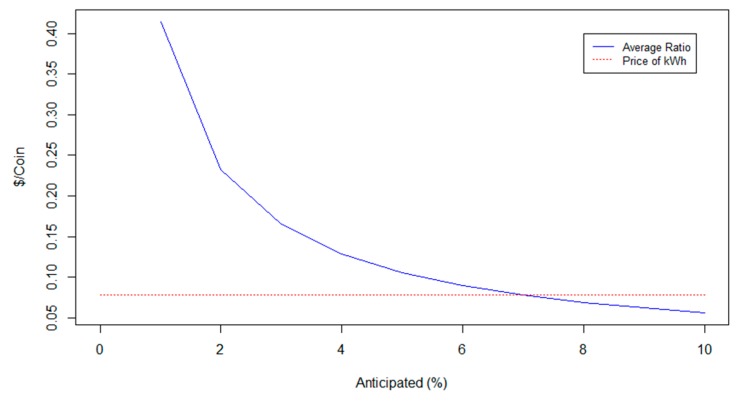
Sensitivity analysis of the ratio according to the percentage of the anticipated savings.

Even if we consider a more pessimistic scenario, where we expected energy savings of 8% and the actual consumption followed the distribution mentioned above, the residents would still benefit financially since the average ratio on that case would have been c_average_ = 0.068 $/coin. In this case, the ratio was lower than the price of a kWh, which is simply leading to less rewards. A sensitivity analysis of how the average ratio changes based on the change of the percentage of the anticipated savings, given that the actual data followed the mentioned distribution, is shown below (Figure 3).

As seen in the above figure, the distribution we followed led to actual savings close to 7% of the anticipated savings, which justify the fact that the average ratio was slightly higher than the price of electrical energy when we expected 6%. However, if the reg the value chosen for the anticipated savings is very pessimistic; therefore, low, the average of the ratio increases disproportionally. This causes our coin to have much more value than the entity it represents making it very dangerous for money laundering schemes, which is something that should be taken into account as the anonymity that blockchain offers in cryptocurrency transactions make it possible for cyber-criminal actions to arise [87]. This proves that choosing realistic values on our constants is of vital importance for the evaluation of the program. Moreover, in case the ratio starts to take values that do not correspond with the basic ideas of the coin, intervention from the authority running the scheme might be necessary in the form of allocating the budget differently or changing some of the constants.

### 4.4. Discussion

**Figure 4:**
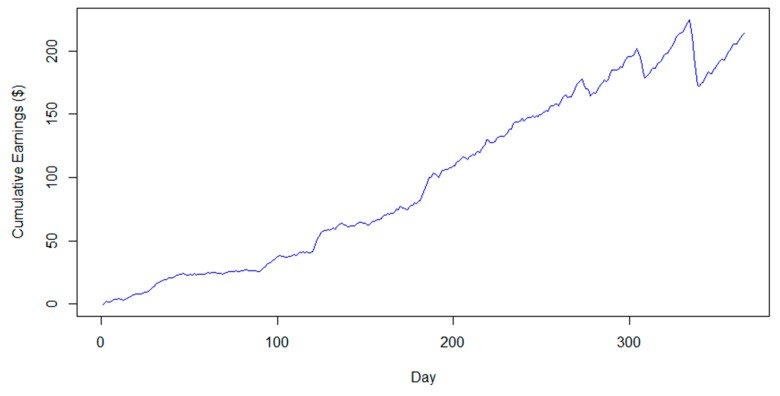
Cumulative monetary gain of a household with six members.

From a financial point of view, with the application of this scheme each household can gain an average of $200 by regulating their consumption, even if we consider the penalties (Figure 4).

**Figure 5:**
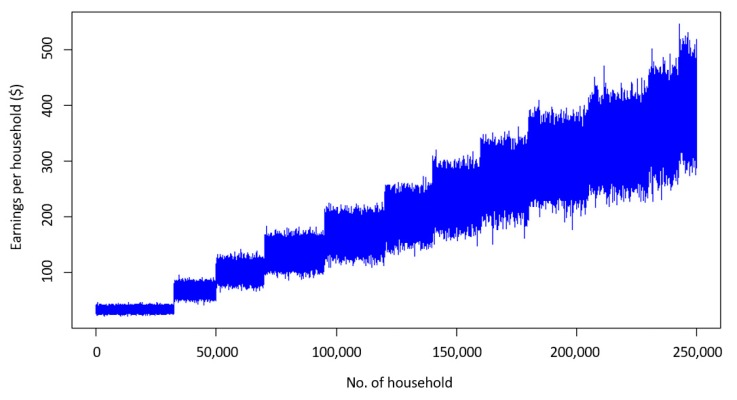
Monetary gain for each household.

**Figure 6:**
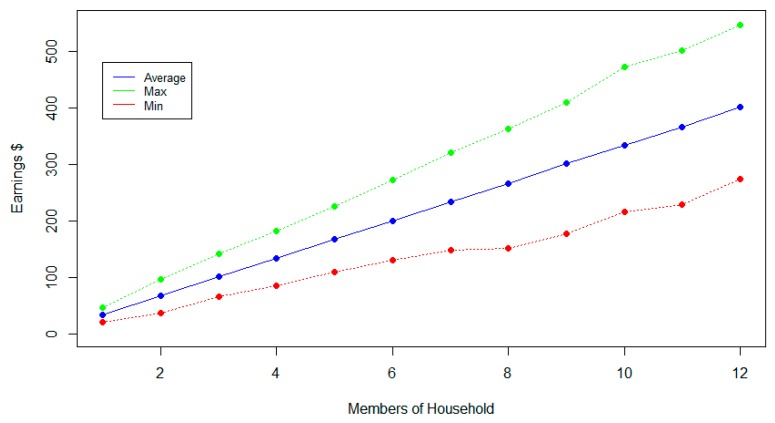
Average monetary gain with minimum and maximum per size.

It is also clear that if a household consumes more energy, which in our case is presented as families with more members, the monetary gain could be larger although there is more variance between households of the same size. Figure 5 presents each one of the monetary gains of the 250,000 households and Figure 6 the average gain with the minimum and the maximum values between households of the same size. The minimum value for the households with eight members was on the same level as the one for seven members. This means that the household that set this minimum value performed poorly compared to the other households of the same category, which is an indicator that in order to experience a financial gain the participants must be committed to saving energy.

From an investing point of view, we calculated the daily return of the ratio using the following formula:

(11) $$Return_{i}\;=\;\frac{Ratio_{i}-Ratio_{i-1}}{Ratio_{i-1}}\;*\;100\%$$

**Figure 7:**
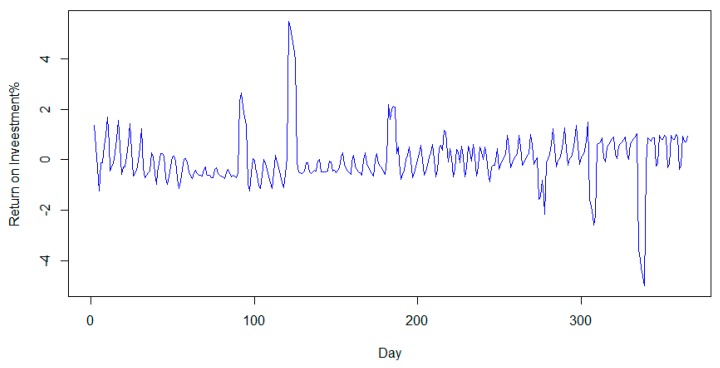
Daily return for ratio.

The results are presented in Figure 7. The average price tends to zero, and the standard deviation tends to 1%. If we consider that the average price can be representative of the expected return and the standard deviation is a metric for risk [91], investing in such an asset is not optimal. This means that financial gain could only be made by someone who saves energy, since simply investing in the currency will not be sufficient in contrast to a minimum variance portfolio. This quality of the currency is important because we want the ratio to be near the actual value of the electrical energy and not to grow indefinitely.

Considering the results from the government’s point of view, this framework is also beneficial, since it can achieve its goal with a relatively low budget. Specifically, a government would have to invest at least 235 million dollars in order to install the required capacity of on shore wind turbines to replace the same amount of energy saved in the simulation for Bahrain. However, the previous amount may vary based on the characteristics of the location, since in our calculations we used a minimum value for the global weighted average in such projects which tends to be 1000 $/kW (IRENA, 2017) [92].

Furthermore, the government may experience another beneficial aspect concerning the approval of the currency. In the worst-case scenario, after the end of the scheme people will cash out their coins to receive the financial benefit that the authority has already accounted. However, if the currency hails the approval of the people, it is possible to be used as an alternate currency. In such a case the scheme could continue in the next years without the necessity for the government to account a budget.

## 5. Conclusions

The integration of energy-currency schemes can have a positive impact on the environment since they result in the reduction of energy consumption. In this paper, we presented the framework for a digital energy currency based on the introduction of energy coins in a smart-energy city, which can be used to offer monetary incentives to every residence, to rationalise each energy consumption. Emphasis was given on the methodology for calculating the ratio of the coin. The idea of rewarding users who are more efficient led to Equation (3), which used the budget for a certain scheme and created a comparison between a target and efficiency achieved both long term and short term.

Using a digital energy currency with a fluctuating value, instead of a traditional monetary reward, aims to make people more aware of the dependence of energy consumption on their behaviour, but also to familiarize them with the idea of energy as a monetary entity without a constant value easing into dynamic pricing [93], and to the new era of the energy market taking advantage of the immense potential of ICT equipment in smart cities. Furthermore, the use of a digital currency addresses liquidity issues, since it may partially replace conventional money in various transactions.

The proposed framework also inspired a pilot scheme which was simulated in the environment of Bahrain. This adaptation demonstrated the manner in which a monetary incentive scheme that uses the energy currency concept may result in the mitigation of the energy consumed in a city. The results proved that, from a financial point of view, such schemes have the potential to be beneficial both for the receiver of the coin and also for the creator. Henceforth, this framework could be used to motivate people to regulate their energy consumption, since they can benefit financially from obtaining energy savings.

The scheme offers insights to policy-makers, in order to promote the proper policies for efficient energy management and the improvement in electricity consumption in the domestic sector.

Further research is necessary to address limitations and issues unveiled in the current study. Initially, in the formulas presented above, the importance of the long-term in relation to the short-term impact should be looked into, and the proper weighting of the respective factors should be included in the formula. Especially for the short-term impact, how many days in the past are taken into account should be thoroughly examined. What is more, smart meters along with historical data should be taken into consideration so as to make the demand prediction models more robust. This causes the framework to be solely implemented in a smart-energy city since it requires the usage of ICT equipment, which is able to monitor in real-time the energy consumption of a building using various smart meters. However, this framework may also be applied in non-smart cities if the necessary equipment is installed, which requires a significant investment that should also be taken into consideration.

Finally, as the study proposes that peer-to-peer transactions could be included, blockchain should be utilised so that transactions can be secure and incorruptible. Different case studies and real implementations of the framework need to be discussed, not only for countries but for smart energy cities, utilities and even cooperatives, as it is already mentioned, to determine the final form of such a currency and the respective benefits.
